# Recent Adaptive Events in Human Brain Revealed by Meta-Analysis of Positively Selected Genes

**DOI:** 10.1371/journal.pone.0061280

**Published:** 2013-04-09

**Authors:** Yue Huang, Chen Xie, Adam Y. Ye, Chuan-Yun Li, Ge Gao, Liping Wei

**Affiliations:** 1 Center for Bioinformatics, State Key Laboratory of Protein and Plant Gene Research, School of Life Sciences, Peking University, Beijing, People's Republic of China; 2 Institute of Molecular Medicine, Peking University, Beijing, People's Republic of China; University of Lausanne, Switzerland

## Abstract

**Background and Objectives:**

Analysis of positively-selected genes can help us understand how human evolved, especially the evolution of highly developed cognitive functions. However, previous works have reached conflicting conclusions regarding whether human neuronal genes are over-represented among genes under positive selection.

**Methods and Results:**

We divided positively-selected genes into four groups according to the identification approaches, compiling a comprehensive list from 27 previous studies. We showed that genes that are highly expressed in the central nervous system are enriched in recent positive selection events in human history identified by intra-species genomic scan, especially in brain regions related to cognitive functions. This pattern holds when different datasets, parameters and analysis pipelines were used. Functional category enrichment analysis supported these findings, showing that synapse-related functions are enriched in genes under recent positive selection. In contrast, immune-related functions, for instance, are enriched in genes under ancient positive selection revealed by inter-species coding region comparison. We further demonstrated that most of these patterns still hold even after controlling for genomic characteristics that might bias genome-wide identification of positively-selected genes including gene length, gene density, GC composition, and intensity of negative selection.

**Conclusion:**

Our rigorous analysis resolved previous conflicting conclusions and revealed recent adaptation of human brain functions.

## Introduction

Humans differ from our closest relative species such as chimpanzees and bonobos in many features including anatomy, physiology, and cognitive functions [1], [2]. Positive selection plays important roles in evolution, especially in creating new phenotypes from ancestral ones [3]–[5]. Identification and analysis of positively-selected genes help us comprehend how unique human features evolved [6]–[9]. The past decades have seen many efforts to explain whether, how and when human Central Nervous System (CNS) evolved, particularly in identifying the events of adaptive evolution in human brain-related genes [10]–[14]. However, previous works have reached conflicting conclusions. Wang *et al*. reported that about 15% positively-selected genes were in the Gene Ontology (GO) category of neuronal functions, indicating overrepresented human brain-related evolution [11], but other works did not find such enrichments in neuronal GO categories [12]–[16]. Nielsen *et al*. also observed that genes under positive selection did not show an excess tendency of brain expression [14].

Genome-wide identification of genes under positive selection has been based on two types of data, inter-species divergence and intra-species polymorphism, either independently or in combination [17]. In divergence-based analyses, the sequences of protein-coding regions from related species were aligned and compared, and the loci with more function-altering changes in one or more lineages are considered to be under positive selection [15], [18]. In contrast, the polymorphism-based approaches, such as F_ST_ and iHS, using population genetic data from a single species, aimed to identify sites that meet the pattern of selective sweep, and contained both the positively-selected targeting allele and the linked neutral alleles [19]–[21]. Recently, many researchers have noted that these two approaches show considerable detection bias: divergence-based approaches focus on detecting fixed adaptive coding changes that occurred near the human-chimp split, while polymorphism-based approaches detect more recent adaptive events in both coding and regulatory regions [3], [4], [6], [22]–[25]. Sabeti *et al*. described this detection bias in detail, and proposed a grouping rule for existing identification approaches [24].

In this paper, we resolved the conflicting conclusions about positive selection of human neuronal genes. By using a meta-analysis approach, we demonstrated that brain-related genes were enriched among positively-selected genes identified by polymorphism-based genomic scan but not divergence-based coding region comparison, suggesting recent brain adaptation in the human lineage. We further showed that most of our observations could not be accounted for by the potential detection biases induced by gene length, gene density, GC composition and intensity of negative selection. Our conclusions were shown to be robust when different datasets, parameters, and analysis pipelines were used.

## Materials and Methods

### Collection of human positively-selected genes and genomic regions

**Figure 1:**
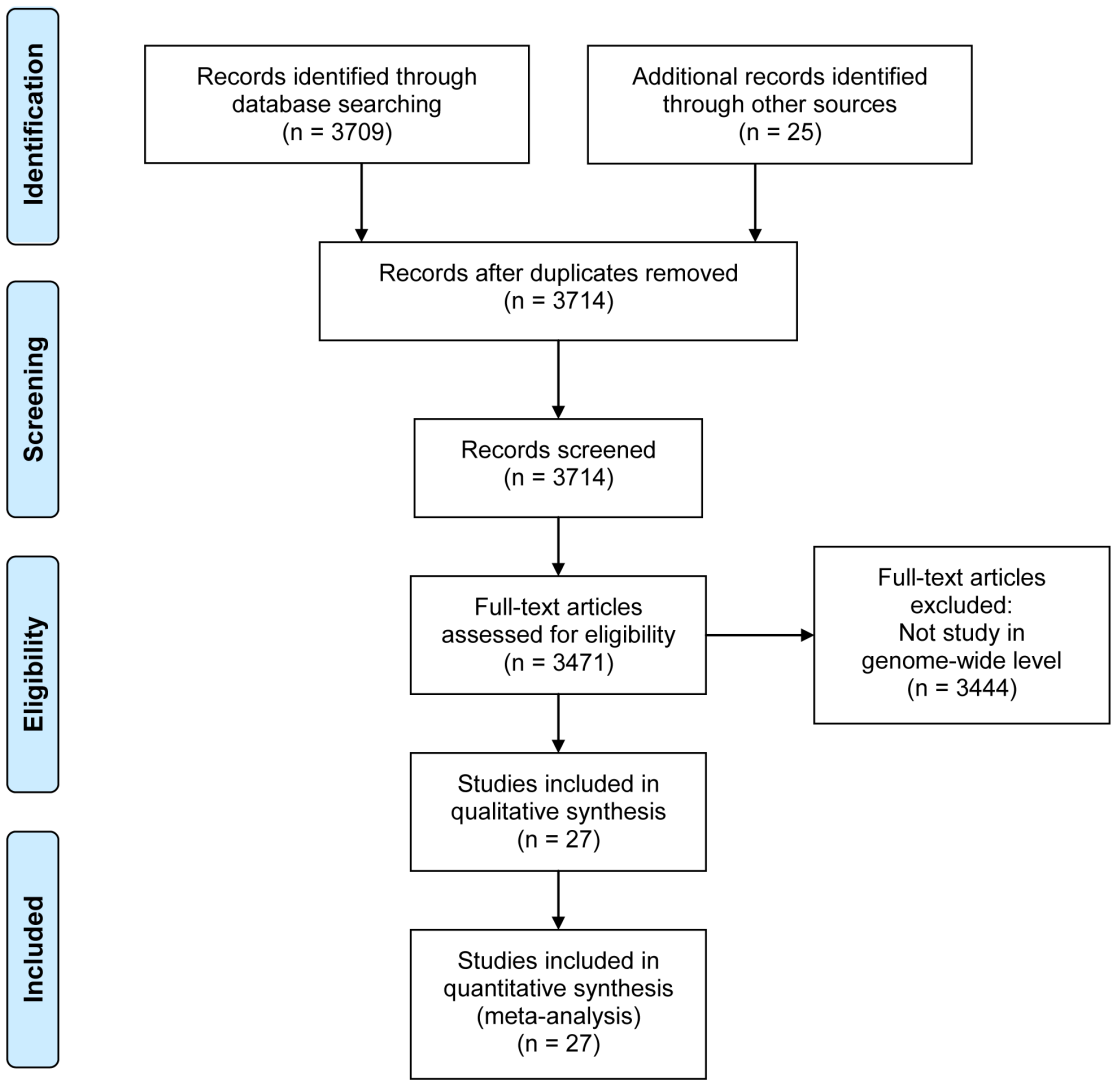
The flow diagram of data collection in accordance with the PRISMA Statement.

We integrated human positively-selected genes identified in previous academic publications, and then grouped them by different identification approaches. To the best of our knowledge, no meta-analysis protocol in studying human positive selection existed, and we developed our meta-analysis pipeline in accordance with the PRISMA Statement (see details in **Text S1**) [26]. In particular, the candidate publication list was retrieved by (i) querying “(positive OR natural OR nonneutral OR adaptive) AND (selection OR evolution) AND genom* AND human” in PubMed with publication date prior to 2011 and (ii) viewing more than 100 review papers about natural selection. Among more than 3700 publications retrieved, twenty-seven publications identified human positively-selected genes at the whole-genomic level were collected (**Figure 1**). We ruled out publications on single-gene analysis in order to avoid ascertainment bias. The gene list was then extracted from these 27 articles, using the identification criteria defined by the original authors (see details in **Text S2**), and unofficial gene symbols were curated by Gene Name Service [27]. These genes were then divided into four groups referring to approaches used to identify positive selection, following the general dividing rule in two reviews [24], [28]: Group 1, a high proportion of function-altering mutations; Group 2, a reduction in genetic diversity; Group 3, a different allele frequency between subpopulations; and Group 4, a long haplotype (**Dataset S1**). Grossman *et al*. developed a composite method integrating multiple signatures of intra-species polymorphism to identify 179 positively-selected genes [21] which were specifically assigned to Group “composite” (**Dataset S1**). We also created a stringent subset of positively-selected genes by collecting only genes identified by two or more studies within each group, and we used it to confirm that our main conclusions still remained.

### Analysis of gene expression data

Two different datasets of expression data, one from mRNA-SEQ and one from cDNA microarray experiments, were analyzed independently to confirm the results.

mRNA-SEQ data was downloaded from http://genes.mit.edu/burgelab/mrna-seq/, which contained transcriptional data of up to 23115 genomic loci in 22 human tissue or cell-line samples, and the RPKM algorithm was applied to evaluate expression levels [29]. The nine tissues obtained from the same source and with comparable reads depth, including adipocyte, brain, heart, liver, lymph node, skeletal muscle, testis, breast, and colon, were used for further analyses. We defined whether the expression of a gene was biased in any tissue by cutting an estimated 2.5% upper-tail of the expression spectrum among all tissues [30]. A gene with expression value in a tissue larger than M+2×MAD would be considered biased-expressed in this tissue, where M and MAD were defined as follows:
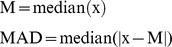
where x indicates the expression values for the corresponding gene among all tissues [31]. To rule out artifacts from thresholds, we later re-set the threshold to “3×median” and obtained similar results (**Figure S1A and B**).

A cDNA microarray dataset GSE1133, which profiled 79 human tissues and cell lines [32], was downloaded from NCBI GEO database [33] and analyzed with R and Bioconductor [34]. Specifically, we used GCRMA for background subtraction, normalization and probe summarization, followed by using Microarray Suite version 5.0 (MAS5; Affymetrix) to call presence or absence. We chose seven tissues which had corresponding mRNA-SEQ data, including adipocyte, brain, heart, liver, lymph node, skeletal muscle, and testis, with two additional tissues, lung and pancreas, to constitute a nine-tissue group. The expression data of 17 CNS regions were also extracted. Probe sets without a MAS5 presence call in any of the nine tissues were excluded. We confirmed that this filtering did not change our conclusions (**Figure S1C**). The same “M+2×MAD” threshold was set to classify whether the gene expression was considered to be biased in a specific tissue. To convert transcriptomic data from probe-set-level into gene-level, the probe-set IDs were converted into Ensembl gene identifiers by using the annotation file of U133A and GNF1H downloaded from BioGPS [35], and a gene with at least one probe-set supporting biased expression in a tissue was considered as biased in that tissue at the gene-level. After probe-to-gene conversion, 16832 genes were annotated with Boolean tags indicating whether the gene's expression was biased in each of the nine tissues, and this gene-level data was used in subsequent analyses.

### Measures of genomic characteristics of human genes

To acquire the information of gene coordinate and structure, the latest Ensembl gene annotation files were downloaded from the UCSC genome browser [36]. Exon, intron and UTR were considered in calculating gene length. Gene density of each gene was measured as the number of genes locating within 100 kb upstream and downstream of a given gene [37]. GC composition was also calculated for each gene together with its 100 kb flanks in both sides. The negative selection intensity on each gene was estimated by dN/dS ratio between human and chimpanzee, downloaded from Ensembl release 69 annotation via BioMart [38].

### Tissue expression enrichment analysis and permutation analysis

We mapped 4357 grouped positively-selected genes into two tissue expression datasets, mRNA-SEQ and cDNA microarray. Genes without expression data were excluded from subsequent statistical tests. A 2×2 contingency table was built for each tissue and each group of positively-selected genes by considering (i) whether a gene was biased expression in certain tissue and (ii) whether it was identified as positively-selected in a certain group. Two-tailed Fisher's exact test was carried out for each contingency table. To adjust Fisher's exact test for multiple testing, the Benjamini and Hochberg FDR corrected P-value was calculated for each test [39]. Since Fisher's exact test is sensitive to the total gene number, we also calculated odds ratios (OR) to evaluate the degree of under-representation or enrichment between positive selection and tissue-biased expression.

The correlation between positive selection and brain expression might be accounted for by the genomic characteristics such as gene length, gene density, GC composition and intensity of negative selection. To explore the influence of such factors, we re-calculate the OR after controlling each factor separately by using the strategy of “permutation in quantiles”, referring to Enard *et al*. [37]. In detail, we first divided the genes into several classes, delimited by the quantiles of one of the four factors, then permutated whether a gene was positively-selected within each class, and finally re-calculated the OR after each permutation. The distributions of permutated log10(OR) were generated by 1000 replicates of permutation, and the mean and standard deviation (s.d.) were calculated for each distribution. **Figure S2 and S3** showed that dividing genes into 15 classes was sufficient in both mRNA-SEQ and cDNA microarray datasets since the mean and s.d. of log10(OR) were not altered much when more classes were used.

### Functional category enrichment analysis and permutation analysis

Functional category enrichment analysis was performed by GOstats [40] in R and Bioconductor environment [34]. The grouped positively-selected genes, which had unique Ensembl ID identified by different approaches, were applied as the input separately, whereas all human Ensembl genes were considered as the background. A hypergeometric test method was applied to calculate the statistical significance of the enriched functional categories of Cellular Component, Biological Process, and Molecular Function. We performed Benjamini and Hochberg FDR correction to adjust for multiple testing [39], and only categories with corrected P-values <0.05 were reported.

To control the influence of gene length, gene density, GC composition, and dN/dS, the same “permutation in quantile” strategy was carried out onto functional category enrichment analysis. Similar to tissue expression enrichment analysis, we divided all the human genes into 15 classes delimited by the quantiles of each factor, permutated whether the gene was belong to the groups of positively selected genes, and finally calculated the ORs for each statistically enriched GO category reported in any of the four groups. The permutated distribution of null hypothesis did not changed much using more classes (**Figure S4**). Because those extremely small GO categories may be vulnerable to the stochastic process of permutation, only categories containing ten or more annotated human genes were reported. For each GO category, the mean and s.d. of the permutated log10(OR) distribution were estimated by 1000 replicates of permutation. Then the one-tailed P-value was calculated as the probability of observing the real log10(OR) or larger from the fitted normal distribution, and was further adjusted by Benjamini and Hochberg FDR correction [39].

## Results

### Integration and grouping of genes and genomic regions under positive selection

After reviewing extensive literature on positive selection, we compiled a list of 4357 genes under positive selection. Except for 179 genes extracted from Grossman *et al*. [21] which were identified by a “composite” method integrating multiple signatures of intra-species polymorphism, the remaining genes were then divided into four groups based on the signatures of positive selection according to Sabeti *et al*. [24] and Hurst *et al*. [28]: Group 1, a high proportion of function-altering mutations; Group 2, a reduction in genetic diversity; Group 3, a different allele frequency between subpopulations; and Group 4, a long haplotype. Group 1 was dominantly based on inter-species divergence, whereas the latter three groups were based on intra-species polymorphism [17]. Group 1 had 1141 human positively-selected genes, and Group 2, 3 and 4 had 1033, 1058 and 1660 genes respectively (**Table S1**). Given an estimated total human gene number of ∼22000 [41], 19.8% of human genes were identified as positively-selected in at least one study, implying that the false positive rate was potentially high [4], [6], [42]-[45].

### Genes highly expressed in the brain show enrichments in recent positive selection

**Figure 2:**
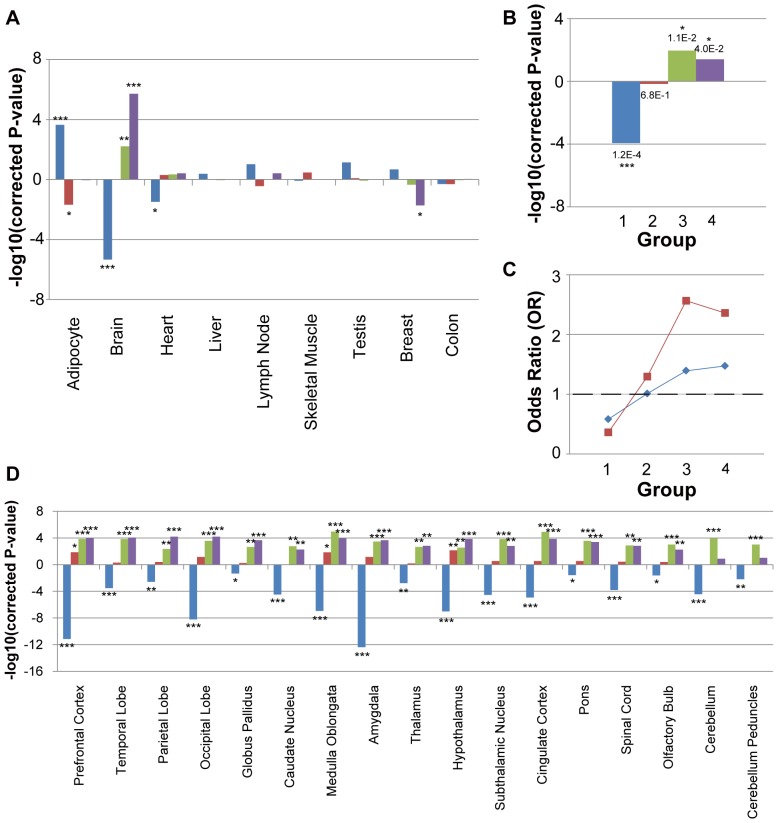
Positively-selected genes of Group 3 and 4 but not Group 1 were highly expressed in brain. (A) Enrichment pattern of positively-selected genes in nine human tissues based on mRNA-SEQ dataset. Blue, red, green and purple bars indicate positively-selected genes in Groups 1, 2, 3 and 4, respectively. The sign of y-axis represents the under-representation (−) or enrichment (+). The bars with significant corrected P-value are marked by asterisks. (B) Brain tissue also shows significant absence (corrected P-value <0.05) for Group 1 and enrichment (corrected P-value <0.05) for Group 3 and 4 based on cDNA microarray dataset. The FDR corrected P-value is labeled on each bar. (C) Using the stringent subset of positively selected genes supported by two or more studies in each group (red square; n  = 85, 79, 11 and 89, respectively), the pattern is even stronger than using all positively selected genes (blue diamond; n  = 1141, 1033, 1058 and 1660, respectively), based on mRNA-SEQ dataset. (D) Fifteen of 17 CNS regions show significant absence (corrected P-value <0.05) for Group 1 genes and enrichment (corrected P-value <0.05) for Group 3 and 4 genes. The two exceptional tissues are cerebellum and cerebellar peduncles.

We analyzed the tissue expression patterns of each of these four gene groups using mRNA-SEQ data of nine tissue samples [29]. Fisher's exact test and subsequent FDR correction [39] were carried out to quantify the correlation between positive selection and tissue-biased expression. As shown in **Figure 2A**, Group 1 positively-selected genes showed enrichment in adipocyte against the background of all known human genes (corrected P-value  = 2.2×10^−4^) but under-representation in brain and heart (corrected P-value  = 4.6×10^−6^ and 3.3×10^−2^, respectively); Group 2 showed under-representation in adipocyte (corrected P-value  = 2.1×10^−2^); Group 3 showed enrichment in brain (corrected P-value  = 6.1×10^−3^); Group 4 showed enrichment in brain (corrected P-value  = 1.9×10^−6^) and under-representation in breast (corrected P-value  = 1.9×10^−2^). This result indicated that some tissues may have been positively-selected within a certain time period in the human lineage. Analysis of cDNA microarray data [32] confirmed a similar pattern for genes with brain-biased expression: Group 1 genes were under-represented in the brain (corrected P-value  = 1.2×10^−4^), whereas Group 3 and 4 genes were enriched in the brain (corrected P-value  = 1.1×10^−2^ and 4.0×10^−2^, respectively) (**Figure 2B**). Group “composite” also showed enrichment of high expression in brain (corrected P-value  = 5.7×10-2 in mRNA-SEQ and 3.4×10-2 in cDNA microarray).

Existing methods to identify genes under positive selection may have relatively high rates of false positives. To confirm the validity of the pattern we observed above, we repeated the analyses using only genes identified by two or more studies to be positively selected in each group. The odds ratio analysis showed that Group 1 genes became more under-represented in brain-biased expression (OR = 0.36 vs. 0.58) and Group 3 and 4 showed stronger enrichment (OR = 2.56 vs. 1.39 and 2.35 vs. 1.47, respectively) (**Figure 2C**).

We also looked at different CNS regions separately and studied positive selection of genes expressed in 17 different CNS regions using the cDNA microarray dataset [32]. Fifteen of 17 regions showed statistical patterns similar to the whole brain, that is, they might have undergone positive selection in recent human evolution (**Figure 2D**). However, two CNS regions, cerebellum and cerebellar peduncles, did not follow this pattern. None of their corrected P-values for Group 4 reached the significance level of 0.05 in these two regions. In fact, cerebellum and its related regions contributed less to human high-level cognitive functions [46]. This implied that recent positive selection events occurred in the vast majority but not all CNS regions.

### Functional categories related to brain also show enrichment in recent positive selection

We next analyzed which functional categories were enriched in each of the four groups of genes under positive selection. As shown in **Table S2**, GO terms of Cellular Components related to the extracellular communication were enriched in Group 1, whereas components related to brain functions such as “synapse”, “synapse part” were enriched in both Group 3 and Group 4. In addition, enriched GO terms of Biological Process further supported the observations: immune-related functions were enriched in Group 1, whereas neuron-related functions, including brain development and synapse communication were enriched in Group 3 and 4 (**Table S2**).

Together, these results showed that enriched functions in Group 1, representing ancient positively-selected coding changes in human history, implied adaptions to unacquainted pathogens. The enriched functions related to brain in Group 3 and 4 indicated that recent adaptive events on human CNS might contribute to the rapid evolution in cognitive functions.

### The brain under-representation and enrichment could not be explained by the detection biases induced by genomic characteristics

**Figure 3:**
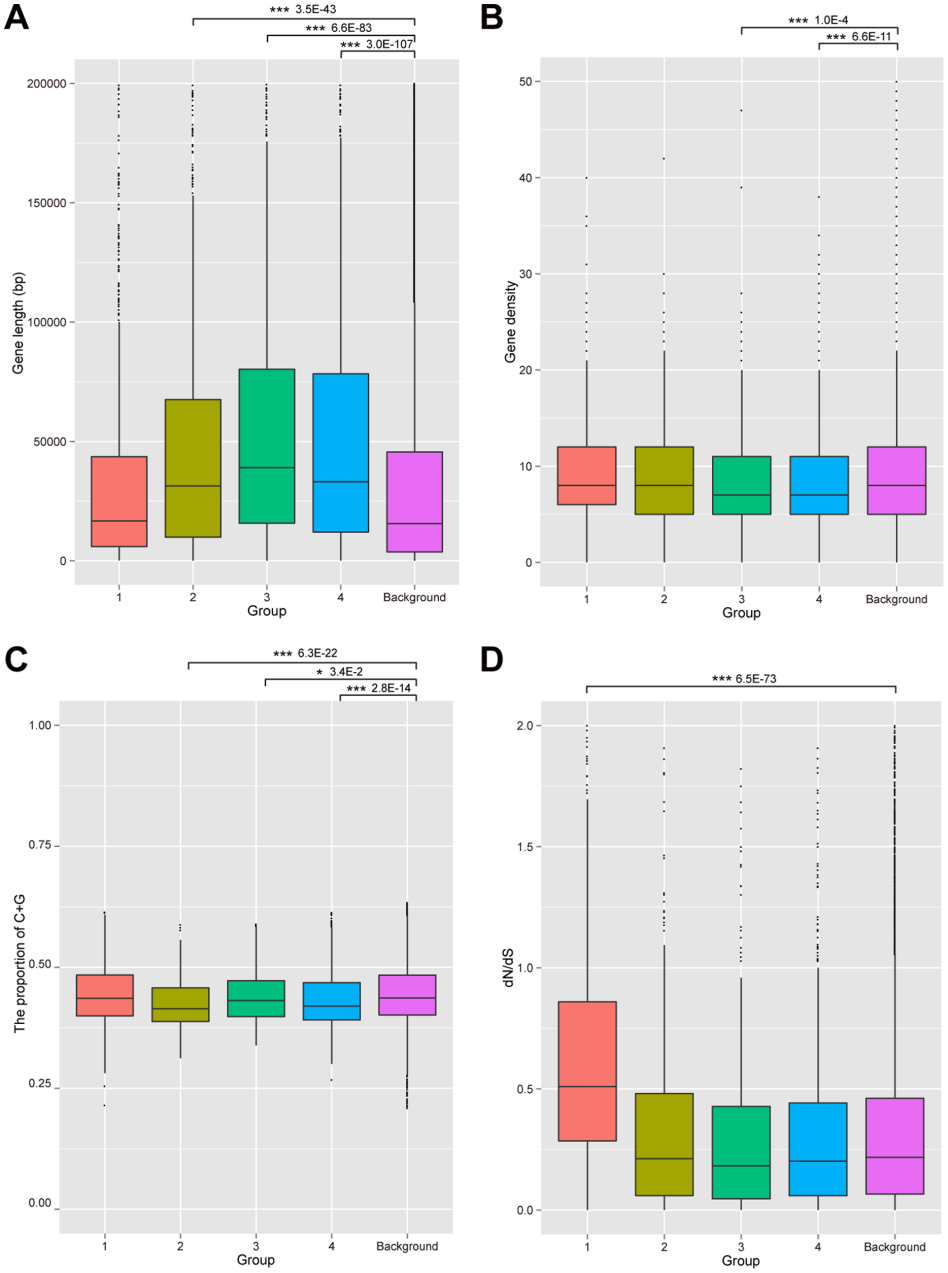
The genomic characteristics varied among groups of positively-selected genes. Boxplots of grouped positively-selected genes by one of the four genomic characteristics: gene length (A), gene density (B), GC composition (C), and dN/dS (D). Wilcoxon two-sample test was carried out between each group pairs, and the P-value was further adjusted by Bonferroni correction. Group 1 genes, primarily identified from inter-species divergence, are distinguished from other three polymorphism-based groups by all four characteristics.

The relatively high false positive rate of existing identification approaches for positively-selected genes raised concerns that the observed brain enrichment and under-representation may be due to some detection biases towards or against brain-related genes. To address this concern, we first analyzed the genomic characteristics of these four groups of positively-selected genes with respect to gene length, gene density, GC composition and intensity of negative selection. Group 1 had almost the same median gene length compared with all human genes, whereas all other groups based on intra-species had significantly longer gene length (**Figure 3A**). Group 3 and 4 had smaller gene density and all groups except Group 1 had less nucleotide composition of C and G than genome background (**Figure 3B and 3C**). Consistent with the signatures of ancient positive selection, Group 1 genes showed a significant excess of dN/dS to genome-wide average; however, this is not the case in other groups of genes (**Figure 3D**). In summary, Group 1 positively-selected genes clearly differed from other intra-species groups in all the four genomic characteristics, suggesting an unnegligible detection bias between inter-species and intra-species identification approaches.

**Figure 4:**
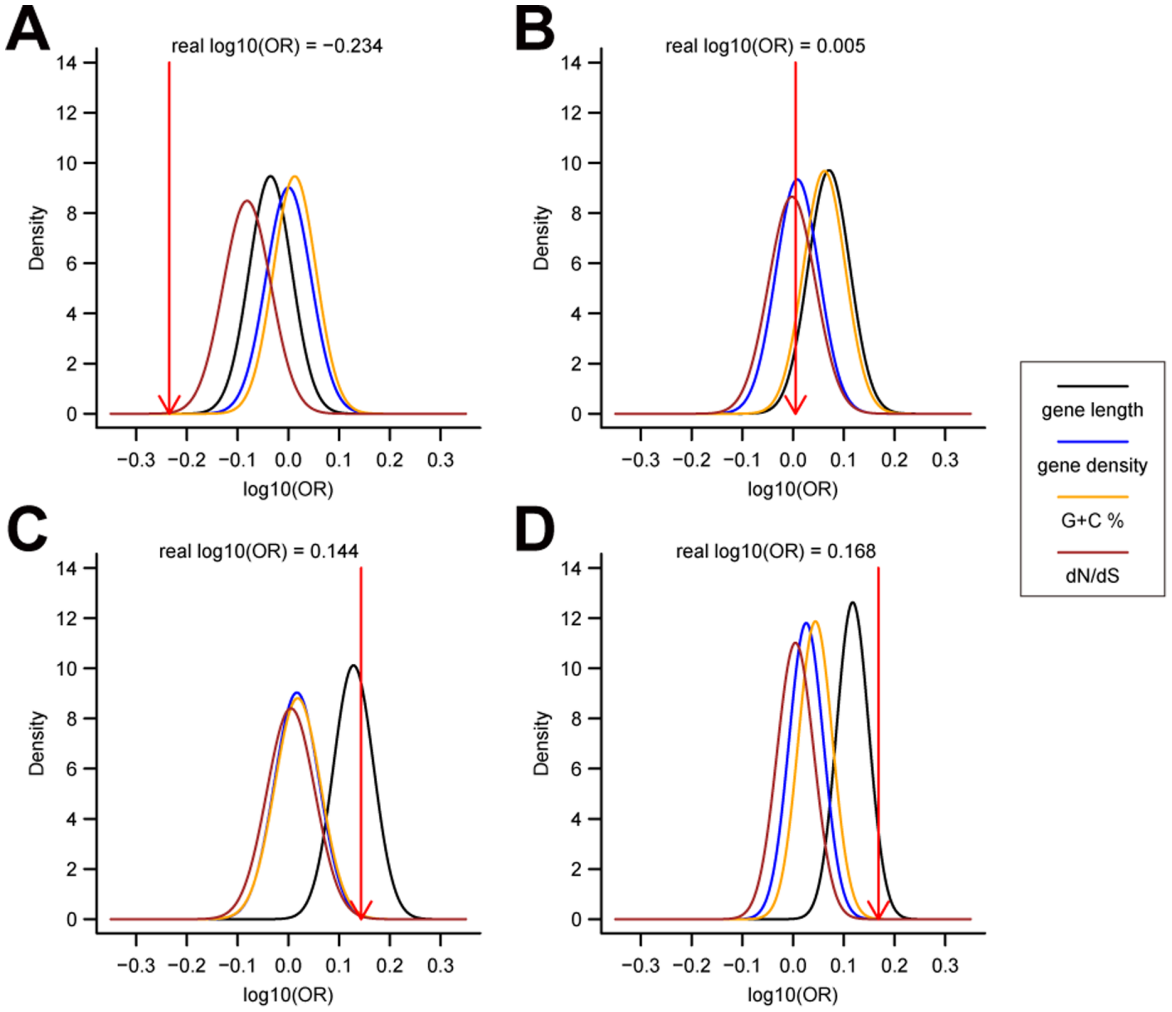
The observed under-representation of brain expression in Group 1 could not be accounted for all the four genomic characteristics, based on mRNA-SEQ dataset. The permutated OR distributions were generated by 1000 replicates after controlling gene length (black), gene density (blue), GC composition (yellow), and dN/dS (brown) for Group 1 to 4 positively selected genes (A-D). Group 1's real OR is significant smaller than expected by chance and it departs from all of the four permutated distributions. Although the real ORs of Group 3 and 4 fall within the 95% confidence interval after controlling gene length, they are larger than the averages of all the four permutated distributions.

We next addressed whether these detection biases could account for the observations that brain-biased expression was under-represented in Group 1 and enriched in Group 3 and 4. To quantify the influence of such detection biases, all human genes were divided into 15 classes by quantiles of one of the four genomic characteristics, and then permutated whether a gene was positively-selected within each class individually (see details in **Materials and Methods**, referring to [37]). Analysis of mRNA-SEQ dataset showed that the observed OR of Group 1 was significantly smaller than expected by chance after controlling any of the four genomic characteristics (**Figure 4A** **)**. On the other hand, although the observed ORs in Group 3 and 4 did not reach the significance level of 0.05 when we controlled gene length, they were larger than the averages in permutated distributions when controlling all four genomic factors (**Figure 4C and 4D**). The same conclusions could also be drawn from cDNA microarray dataset (**Figure S5**). These results suggested that the under-representation of brain-expressed genes in Group 1 could not be explained by the influence of any of the four factors, whereas the observed enrichment in Group 3 and 4 was, to some extent, affected by the gene length bias. Nevertheless, it still could be seen that the real enrichment level was higher than expected by chance, and more analysis might be required to verify the recent positive selection in human brain evolution.

**Figure 5:**
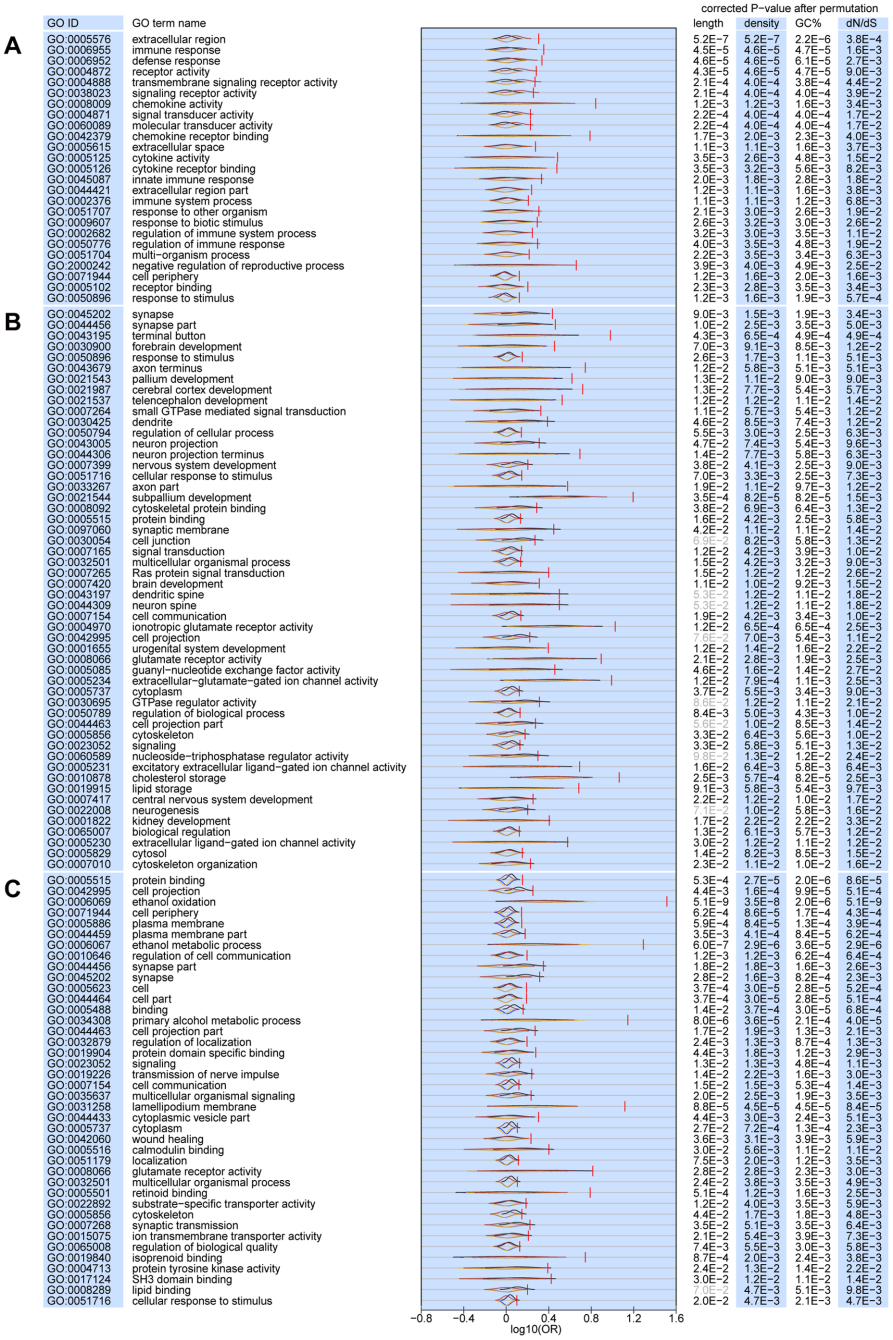
All the immune-related and most of the brain-related GO terms remain significant in Group 1 and Group 3 and 4 after controlling the influence of four genomic characteristics. The log10-transformed real OR and four permutated OR distributions after controlling the factor of gene length (black, above), gene density (blue, below), GC composition (yellow, below), and dN/dS (blue, above) were plotted for each statistically enriched GO term in Group 1 (A), Group 3 (B), and Group 4 (C). The FDR corrected P-values were also calculated after controlling those four factors separately, and those corrected P-values larger than 0.05 were marked in grey. All the immune-related GO terms in Group 1 and most of the brain-related GO terms in Group 3 and 4 remain statistically significant.

To address this issue, we further applied the same “permutation in quantiles” strategy to functional categories enrichment analysis. **Figure 5A** showed that all the immune-related GO terms remained significant in Group 1 genes even after we controlled those four genomic characteristics. Although gene length could account for the enrichment of some previously-reported GO terms in Group 3 and 4, they were still significantly enriched in most of the brain-related GO terms reported (**Figure 5B and 5C**). This indicated that recent positive selection had indeed occurred in those genes contributing to some certain brain-related functions, even after controlling the potential detection biases induced by all the four genomic characteristics.

## Discussion

We present a clearer picture of positive selection in the human lineage, with the conclusion that major evolutionary changes in different tissues and different functional groups occurred predominantly at particular time periods, some near the chimpanzee-human divergence and others much more recently. Our analyses of expression patterns and functional categories consistently support recent adaptation in the human brain. After controlling the notable detection biases induced by four genomic characteristics, we could still observe an excess of recent brain evolution from expression data, and these results were further supported by functional category enrichment analysis. Our results provide explanations of previously conflicting results about the evolution of brain-related positively-selected genes.

Previous studies based on inter-species divergence have reported that brain-related GO categories were not enriched in human positive selection [12]–[16], and they are consistent with our finding that Group 1 positively-selected genes indeed showed under-representation of brain-biased expression. It has been noticed that such divergence-based approaches focused on searching signals of positive selection in protein-coding regions and lacked the power to detect adaptive changes in regulatory regions. Notably, Haygood *et al*. had reported brain-related enrichment by scanning the evolutionary substitutes in promoter regions between human and chimpanzee [47]. Although we have shown that divergence-based identification approaches seem not to suffer a lot from genomic context, we cannot exclude the possibility that other factors might contribute to the under-representation of brain-related genes, such as more frequent evolutionary changes occurred in regulatory regions of brain-related genes instead of their protein-coding regions.

One may raise the concern that our enrichment analysis might have more power to detect brain-related processes if brain-related genes are with a larger number and are well-annotated in GO database. Here, we addressed this concern from two aspects. Firstly, we demonstrated that the number of brain-expressed genes (n = 3188) was not the most among all the tissues we used (n ranges from 1201 to 5529); in addition, GO annotation did not show preference for brain-expressed genes, compared with all human genes (87% vs. 86%). Secondly, in addition to Fisher's exact test, we used OR as an alternative estimate which is less sensitive to the number of input gene, and our findings of brain enrichment pattern remained unchanged (**Figure 2C** **and** **Table S3**). As a result, the observed brain enrichment is unlikely to be led by the difference of statistical power.

It should be noted again that existing genome-wide approaches to identify positively-selected genes have relatively high false positive rate; thus the statistical signals of under-representation or enrichment might be diluted, which made us potentially missed some true signals. This might be an explanation why we could not find any enrichment signals in Group 2 positively-selected genes. Our results also emphasize that the characteristics of genomic context should be considered seriously when we interpret the result generated from such genome-wide scans. For instance, the genes with larger gene size would have more chance to overlap with any windows in genomic scans. The lessons learned from this study might inform future genome-wide studies. Strictly speaking, the genes under positive selection that we analyzed here are in fact genes under “putative” positive selection.

After controlling gene length, the permutation results of both tissue expression and functional categories enrichment analyses suggested an excess of brain-related adaptation in Group 3 and 4, whereas the significance level reached 0.05 in many neuronal GO terms but not in brain-biased expression. This raised a possibility that, if the recent adaptive brain evolution had occurred only in some particular brain functions, the enrichment signal might be diluted when globally considering all brain-expressed genes. The hierarchical GO system provided us an opportunity to test the enrichment in many sub-classified gene functions, which may lead to higher sensitivity to detect the enrichment signal of positive selection.

In this study, we primarily addressed the conflicts about adaptation in the human brain. However, genes involved in testis and spermatogenesis were also reported having experienced adaptive evolution identified by inter-species divergence [14], [23]. We found a weak enrichment of testis-biased expression in Group 1 from mRNA-SEQ dataset (corrected P-value = 0.07 and OR = 1.19), but it was not supported by cDNA microarray dataset (corrected P-value = 0.69 and OR = 0.90). The discordance between two expression datasets asks for further studies in the future. We did not find testis enrichment in the latter three groups from either mRNA-SEQ or cDNA microarray dataset. Consistent with the previous study [12], we found that immune-related functions were enriched only in Group 1 positively-selected genes, but not in the latter three groups. By developing a method to identify adaptive evolution at single SNP resolution, Fumagalli *et al*. found that immune-related functions were involved in subpopulation divergence and adaptation [48]. This discovery supports our point that reducing the false positive rate of identification approaches might provide more insights into human evolution.

With the advances of next-generation sequencing, the statistical power to detect events of positive selection will be benefited when more primate genomes are sequenced and individual human genomes are re-sequenced at greater coverage [24]. Eventually, continuous progress in this area will enable us to decode a clearer picture of human evolution.

## Supporting Information

Figure S1 **Enrichment patterns of human brain tissue from expression data with alternative parameters and analysis pipelines.** The enrichment patterns were generated from mRNA-SEQ (A) and cDNA microarray (B, C) datasets. The x-axis represents four groups of positively-selected genes and the sign of y-axis represents the under-representation (−) or enrichment (+). The bars with significant corrected P-value are marked by asterisks. In panel A and panel B, the threshold of biased-expressed was “3×median”, instead of “median+2×MAD”. In panel C, the cDNA microarray data was pretreated without filtering out absent probe sets in MAS5 presence call of all nine tissues. The absence (corrected P-value <0.05) for Group 1 positively-selected genes and enrichment (corrected P-value <0.05) for Group 3 and 4 in brain tissue remain in all panels. This result implies that our result is robust under varied datasets, thresholds and analysis pipelines.
(TIF)

Figure S2 **Performance of “permutation in quantiles” in tissue expression enrichment analysis, based on mRNA-SEQ dataset.** The OR distribution was generated by 1000 replicates of permutation with varied number of quantiles. Black, blue and orange lines denotes the mean, mean±s.d. and 95% confidence intervals for each permutated log10(OR) distribution while the red line denotes the observed log10(OR). Row 1–4 represent Group 1, 2, 3 and 4 positively selected genes, and column 1–4 represent controlling the genomic characteristics of gene length, gene density, GC composition or dN/dS. The permutated OR distribution remain almost the same when the number of classes is larger than 15.
(TIF)

Figure S3 **Performance of “permutation in quantiles” in tissue expression enrichment analysis, based on cDNA microarray dataset.** The OR distribution was generated by 1000 replicates of permutation with varied number of quantiles. Black, blue and orange lines denotes the mean, mean±s.d. and 95% confidence intervals for each permutated log10(OR) distribution while the red line denotes the observed log10(OR). Row 1–4 represent Group 1, 2, 3 and 4 positively selected genes, and column 1–4 represent controlling the genomic characteristics of gene length, gene density, GC composition or dN/dS. The permutated OR distribution remain almost the same when the number of classes is larger than 15.
(TIF)

Figure S4 **Performance of “permutation in quantiles” in functional category enrichment analysis.** The mean (A) and s.d. (B) of each significant GO term in Group 1 was generated by 1000 replicates of permutation with varied number of classes delimited by the factor of dN/dS. The mean and s.d. of permutated OR distribution is not altered much when the number of class is larger than 15.
(TIF)

Figure S5 **The observed under-representation of brain expression in Group 1 could not be accounted for all the four genomic characteristics, based on cDNA microarray dataset.** The permutated OR distributions were generated by 1000 replicates after controlling gene length (black), gene density (blue), GC composition (yellow), and dN/dS (brown) for Group 1 to 4 positively selected genes (A–D). Group 1's real OR is significant smaller than expected by chance and it departs from all of the four permutated distributions. Although the real ORs of Group 3 and 4 fall within the 95% confidence interval after controlling gene length, they are larger than the averages of all the four permutated distributions.
(TIF)

Table S1 **Summary of grouped positively-selected genes by different approaches.**
(DOCX)

Table S2 **Enriched GO terms in different groups of human positively-selected genes.**
(DOCX)

Table S3 **Odds ratios of brain-biased expression enrichment analysis for each groups of positively-selected genes.**
(DOCX)

Text S1 **The checklist of the PRISMA Statement.**
(DOCX)

Text S2 **The detailed description of identification approaches for 27 literatures identifying human positively selected genes.**
(DOCX)

Dataset S1 **The detailed information about human positively-selected genes in our collection.**
(XLSX)

## References

[1] Carroll SB (2003) Genetics and the making of Homo sapiens. Nature 422: 849–857. 12712196 10.1038/nature01495

[2] Roth G, Dicke U (2005) Evolution of the brain and intelligence. Trends Cogn Sci 9: 250–257. 15866152 10.1016/j.tics.2005.03.005

[3] Nielsen R, Hellmann I, Hubisz M, Bustamante C, Clark AG (2007) Recent and ongoing selection in the human genome. Nat Rev Genet 8: 857–868. 17943193 10.1038/nrg2187 PMC2933187

[4] Biswas S, Akey JM (2006) Genomic insights into positive selection. Trends Genet 22: 437–446. 16808986 10.1016/j.tig.2006.06.005

[5] Enard W, Przeworski M, Fisher SE, Lai CSL, Wiebe V, et al (2002) Molecular evolution of FOXP2, a gene involved in speech and language. Nature 418: 869–872. 12192408 10.1038/nature01025

[6] Akey JM (2009) Constructing genomic maps of positive selection in humans: Where do we go from here? Genome Res 19: 711–722. 19411596 10.1101/gr.086652.108 PMC3647533

[7] Gilbert SL, Dobyns WB, Lahn BT (2005) Genetic links between brain development and brain evolution. Nat Rev Genet 6: 581–590. 15951746 10.1038/nrg1634

[8] Zhang J, Webb DM, Podlaha O (2002) Accelerated Protein Evolution and Origins of Human-Specific Features: FOXP2 as an Example. Genetics 162: 1825–1835. 12524352 10.1093/genetics/162.4.1825 PMC1462353

[9] Tang BL (2006) Molecular genetic determinants of human brain size. Biochem Biophys Res Commun 345: 911–916. 16716254 10.1016/j.bbrc.2006.05.040

[10] Wang H-Y, Chien H-C, Osada N, Hashimoto K, Sugano S, et al (2007) Rate of Evolution in Brain-Expressed Genes in Humans and Other Primates. PLoS Biol 5: e13. 17194215 10.1371/journal.pbio.0050013 PMC1717015

[11] Wang ET, Kodama G, Baldi P, Moyzis RK (2006) Global landscape of recent inferred Darwinian selection for Homo sapiens. Proc Natl Acad Sci U S A 103: 135–140. 16371466 10.1073/pnas.0509691102 PMC1317879

[12] Consortium TCSaA (2005) Initial sequence of the chimpanzee genome and comparison with the human genome. Nature 437: 69–87. 16136131 10.1038/nature04072

[13] Bustamante CD, Fledel-Alon A, Williamson S, Nielsen R, Hubisz MT, et al (2005) Natural selection on protein-coding genes in the human genome. Nature 437: 1153–1157. 16237444 10.1038/nature04240

[14] Nielsen R, Bustamante C, Clark AG, Glanowski S, Sackton TB, et al (2005) A Scan for Positively Selected Genes in the Genomes of Humans and Chimpanzees. PLoS Biol 3: e170. 15869325 10.1371/journal.pbio.0030170 PMC1088278

[15] Arbiza L, Dopazo J, Dopazo H (2006) Positive Selection, Relaxation, and Acceleration in the Evolution of the Human and Chimp Genome. PLoS Comput Biol 2: e38. 16683019 10.1371/journal.pcbi.0020038 PMC1447656

[16] Bakewell MA, Shi P, Zhang J (2007) More genes underwent positive selection in chimpanzee evolution than in human evolution. Proc Natl Acad Sci U S A 104: 7489–7494. 17449636 10.1073/pnas.0701705104 PMC1863478

[17] Jensen JD, Wong A, Aquadro CF (2007) Approaches for identifying targets of positive selection. Trends Genet 23: 568–577. 17959267 10.1016/j.tig.2007.08.009

[18] Clark AG, Glanowski S, Nielsen R, Thomas PD, Kejariwal A, et al (2003) Inferring Nonneutral Evolution from Human-Chimp-Mouse Orthologous Gene Trios. Science 302: 1960–1963. 14671302 10.1126/science.1088821

[19] Voight BF, Kudaravalli S, Wen X, Pritchard JK (2006) A Map of Recent Positive Selection in the Human Genome. PLoS Biol 4: e72. 16494531 10.1371/journal.pbio.0040072 PMC1382018

[20] Akey JM, Zhang G, Zhang K, Jin L, Shriver MD (2002) Interrogating a High-Density SNP Map for Signatures of Natural Selection. Genome Res 12: 1805–1814. 12466284 10.1101/gr.631202 PMC187574

[21] Grossman SR, Shylakhter I, Karlsson EK, Byrne EH, Morales S, et al (2010) A Composite of Multiple Signals Distinguishes Causal Variants in Regions of Positive Selection. Science 327: 883–886. 20056855 10.1126/science.1183863

[22] Zhai W, Nielsen R, Slatkin M (2009) An Investigation of the Statistical Power of Neutrality Tests Based on Comparative and Population Genetic Data. Mol Biol Evol 26: 273–283. 18922762 10.1093/molbev/msn231 PMC2727393

[23] Kosiol C, Vinar T, Fonseca RRd, Hubisz MJ, Bustamante CD, et al (2008) Patterns of Positive Selection in Six Mammalian Genomes. PLoS Genet 4: e1000144. 18670650 10.1371/journal.pgen.1000144 PMC2483296

[24] Sabeti PC, Schaffner SF, Fry B, Lohmueller J, Varilly P, et al (2006) Positive Natural Selection in the Human Lineage. Science 312: 1614–1620. 16778047 10.1126/science.1124309

[25] Moreno-Estrada A, Tang K, Sikora M, Marques-Bonet T, Casals F, et al (2009) Interrogating 11 Fast-Evolving Genes for Signatures of Recent Positive Selection in Worldwide Human Populations. Mol Biol Evol 26: 2285–2297. 19578157 10.1093/molbev/msp134

[26] Moher D, Liberati A, Tetzlaff J, Altman DG (2009) Group TP (2009) Preferred Reporting Items for Systematic Reviews and Meta-Analyses: The PRISMA Statement. PLoS Medicine 6: e1000097. 19621072 10.1371/journal.pmed.1000097 PMC2707599

[27] Lin K-T, Liu C-H, Chiou J-J, Tseng W-H, Lin K-L, et al.. (2007) Gene name service: no-nonsense alias resolution service for Homo Sapiens genes. Silicon Valley,CA,USA.pp. 185–188.

[28] Hurst LD (2009) Genetics and the understanding of selection. Nat Rev Genet 10: 83–93. 19119264 10.1038/nrg2506

[29] Wang ET, Sandberg R, Luo S, Khrebtukova I, Zhang L, et al (2008) Alternative isoform regulation in human tissue transcriptomes. Nature 456: 470–476. 18978772 10.1038/nature07509 PMC2593745

[30] Daszykowski M, Kaczmarek K, Heyden YV, Walczak B (2007) Robust statistics in data analysis - A review: Basic concepts. Chemometrics and Intelligent Laboratory Systems 85: 203–219.

[31] Chung N, Zhang XD, Kreamer A, Locco L, Kuan P-F, et al (2008) Median Absolute Deviation to Improve Hit Selection for Genome-Scale RNAi Screens. J Biomol Screen 13: 149–158. 18216396 10.1177/1087057107312035

[32] Su AI, Wiltshire T, Batalov S, Lapp H, Ching KA, et al (2004) A gene atlas of the mouse and human protein-encoding transcriptomes. Proc Natl Acad Sci U S A 101: 6062–6067. 15075390 10.1073/pnas.0400782101 PMC395923

[33] Barrett T, Troup DB, Wilhite SE, Ledoux P, Rudnev D, et al (2009) NCBI GEO: archive for high-throughput functional genomic data. Nucleic Acids Res 37: D885–890. 18940857 10.1093/nar/gkn764 PMC2686538

[34] Gentleman RC, Carey VJ, Bates DM, Bolstad B, Dettling M, et al (2004) Bioconductor: open software development for computational biology and bioinformatics. Genome Biol 5: R80. 15461798 10.1186/gb-2004-5-10-r80 PMC545600

[35] Wu C, Orozco C, Boyer J, Leglise M, Goodale J, et al (2009) BioGPS: an extensible and customizable portal for querying and organizing gene annotation resources. Genome Biol 10: R130. 19919682 10.1186/gb-2009-10-11-r130 PMC3091323

[36] Dreszer TR, Karolchik D, Zweig AS, Hinrichs AS, Raney BJ, et al (2012) The UCSC Genome Browser database: extensions and updates 2011. Nucleic Acids Res 2012: D918–D923. 10.1093/nar/gkr1055 PMC3245018 22086951

[37] Enard D, Depaulis F, Crollius HR (2010) Human and Non-Human Primate Genomes Share Hotspots of Positive Selection. PLoS Genet 6: e1000840. 20140238 10.1371/journal.pgen.1000840 PMC2816677

[38] Flicek P, Amode MR, Barrell D, Beal K, Brent S, et al (2012) Ensembl 2012. Nucleic Acids Res 40: D84–D90. 22086963 10.1093/nar/gkr991 PMC3245178

[39] Benjamini Y, Hochberg Y (1995) Controlling the False Discovery Rate: A Practical and Powerful Approach to Multiple Testing. J R Statist Soc 57: 289–300.

[40] Falcon S, Gentleman R (2007) Using GOstats to test gene lists for GO term association. Bioinformatics 23: 257–258. 17098774 10.1093/bioinformatics/btl567

[41] Pertea M, Salzberg SL (2010) Between a chicken and a grape: estimating the number of human genes. Genome Biol 11: 206. 20441615 10.1186/gb-2010-11-5-206 PMC2898077

[42] Teshima KM, Coop G, Przeworski M (2006) How reliable are empirical genomic scans for selective sweeps? . Genome Res Vol 16: 702–712. 10.1101/gr.5105206 PMC1473181 16687733

[43] Boyko AR, Williamson SH, Indap AR, Degenhardt JD, Hernandez RD, et al (2008) Assessing the Evolutionary Impact of Amino Acid Mutations in the Human Genome. PLoS Genet 4: e1000083. 18516229 10.1371/journal.pgen.1000083 PMC2377339

[44] Mallick S, Gnerre S, Muller P, Reich D (2009) The difficulty of avoiding false positives in genome scans for natural selection. Genome Res Vol. 19: 922–933. 10.1101/gr.086512.108 PMC2675981 19411606

[45] Fletcher W, Yang Z (2010) The Effect of Insertions, Deletions, and Alignment Errors on the Branch-Site Test of Positive Selection. Mol Biol Evol Vol. 27: 2257–2267. 10.1093/molbev/msq115 20447933

[46] Rakic P (2009) Evolution of the neocortex: a perspective from developmental biology. Nat Rev Neurosci 10: 724–735. 19763105 10.1038/nrn2719 PMC2913577

[47] Haygood R, Fedrigo O, Hanson B, Yokoyama K-D, Wray GA (2007) Promoter regions of many neural- and nutrition-related genes have experienced positive selection during human evolution. Nat Genet Vol 39. 10.1038/ng2104 17694055

[48] Fumagalli M, Sironi M, Pozzoli U, Ferrer-Admettla A, Pattini L, et al (2011) Signatures of Environmental Genetic Adaptation Pinpoint Pathogens as the Main Selective Pressure through Human Evolution. PLoS Genet 7: e1002355. 22072984 10.1371/journal.pgen.1002355 PMC3207877

