# Supplementary figures and images for: Recent Adaptive Events in Human Brain Revealed by Meta-Analysis of Positively Selected Genes
Source: PLoS One. 2013 Apr 9;8(4):e61280. doi: 10.1371/journal.pone.0061280 (PMC3622023; doi:10.1371/journal.pone.0061280)

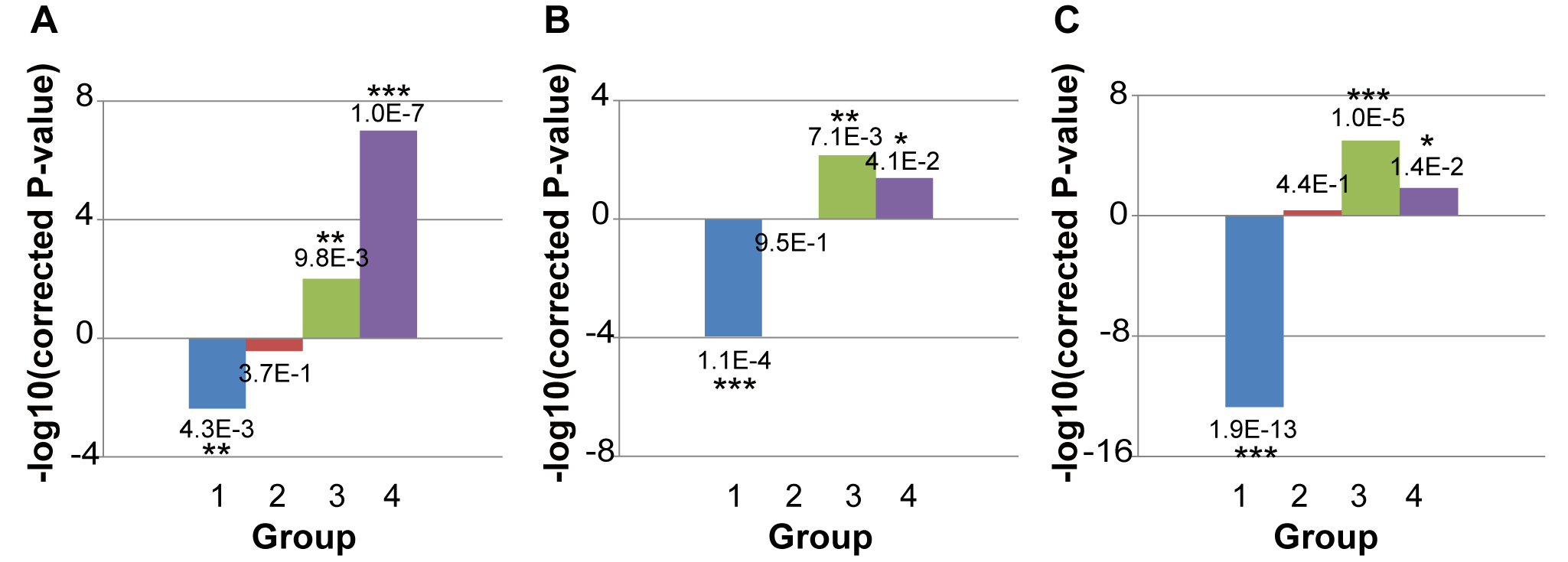

Supplement: Figure S1 — Enrichment patterns of human brain tissue from expression data with alternative parameters and analysis pipelines. The enrichment patterns were generated from mRNA-SEQ (A) and cDNA microarray (B, C) datasets. The x-axis represents four groups of positively-selected genes and the sign of y-axis represents the under-representation (−) or enrichment (+). The bars with significant corrected P-value are marked by asterisks. In panel A and panel B, the threshold of biased-expressed was “3×median”, instead of “median+2×MAD”. In panel C, the cDNA microarray data was pretreated without filtering out absent probe sets in MAS5 presence call of all nine tissues. The absence (corrected P-value <0.05) for Group 1 positively-selected genes and enrichment (corrected P-value <0.05) for Group 3 and 4 in brain tissue remain in all panels. This result implies that our result is robust under varied datasets, thresholds and analysis pipelines. (TIF) [file pone.0061280.s001.tif]

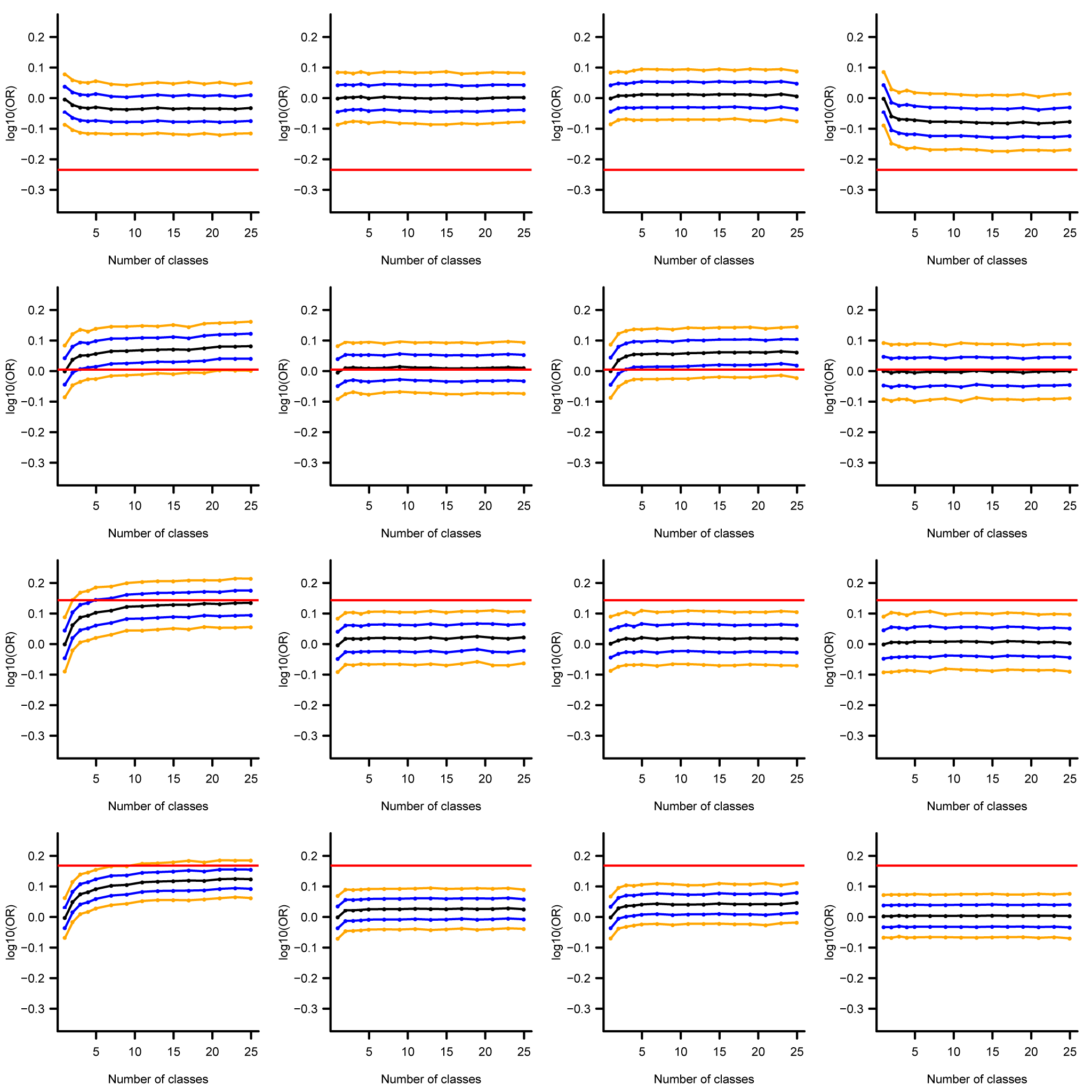

Supplement: Figure S2 — Performance of “permutation in quantiles” in tissue expression enrichment analysis, based on mRNA-SEQ dataset. The OR distribution was generated by 1000 replicates of permutation with varied number of quantiles. Black, blue and orange lines denotes the mean, mean±s.d. and 95% confidence intervals for each permutated log10(OR) distribution while the red line denotes the observed log10(OR). Row 1–4 represent Group 1, 2, 3 and 4 positively selected genes, and column 1–4 represent controlling the genomic characteristics of gene length, gene density, GC composition or dN/dS. The permutated OR distribution remain almost the same when the number of classes is larger than 15. (TIF) [file pone.0061280.s002.tif]

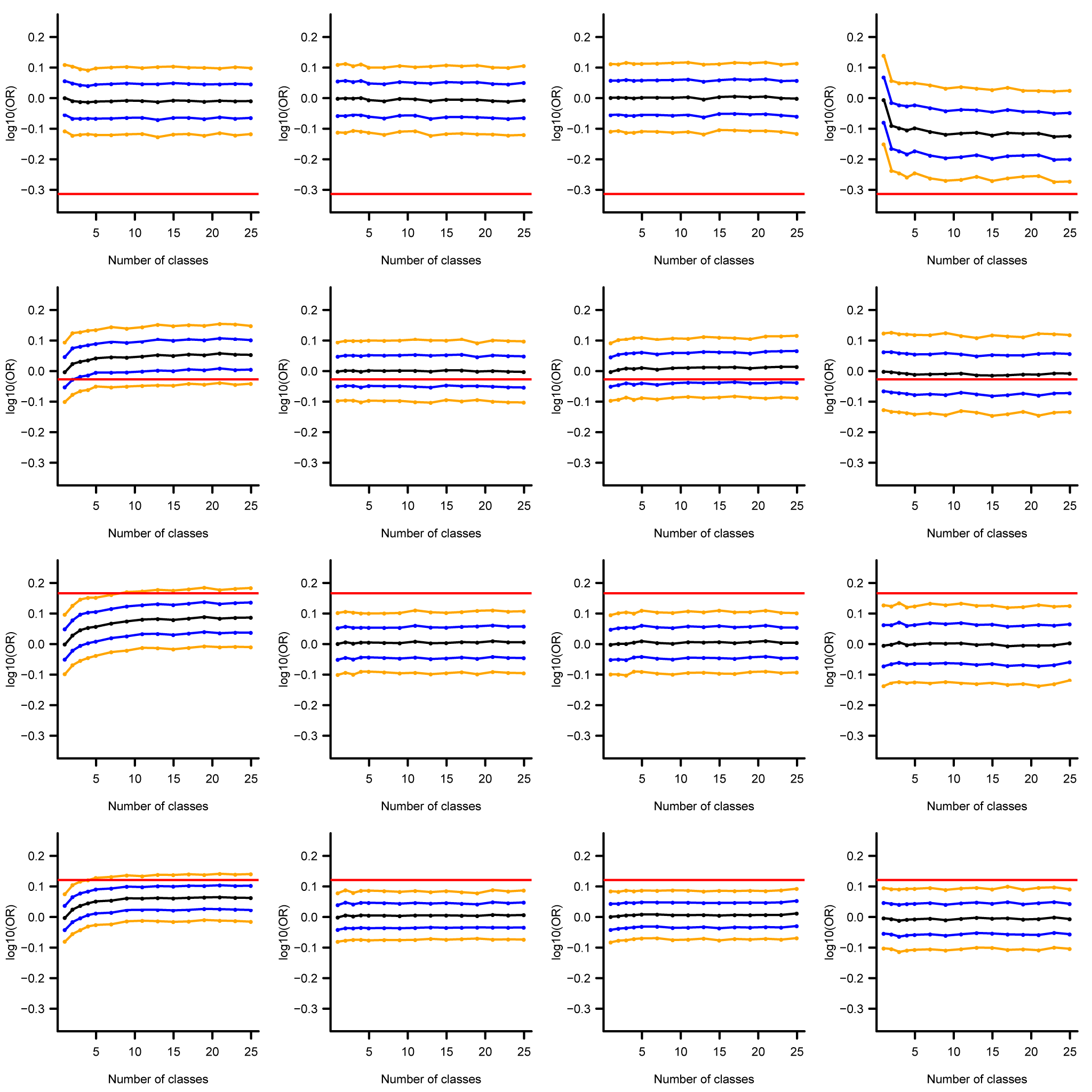

Supplement: Figure S3 — Performance of “permutation in quantiles” in tissue expression enrichment analysis, based on cDNA microarray dataset. The OR distribution was generated by 1000 replicates of permutation with varied number of quantiles. Black, blue and orange lines denotes the mean, mean±s.d. and 95% confidence intervals for each permutated log10(OR) distribution while the red line denotes the observed log10(OR). Row 1–4 represent Group 1, 2, 3 and 4 positively selected genes, and column 1–4 represent controlling the genomic characteristics of gene length, gene density, GC composition or dN/dS. The permutated OR distribution remain almost the same when the number of classes is larger than 15. (TIF) [file pone.0061280.s003.tif]

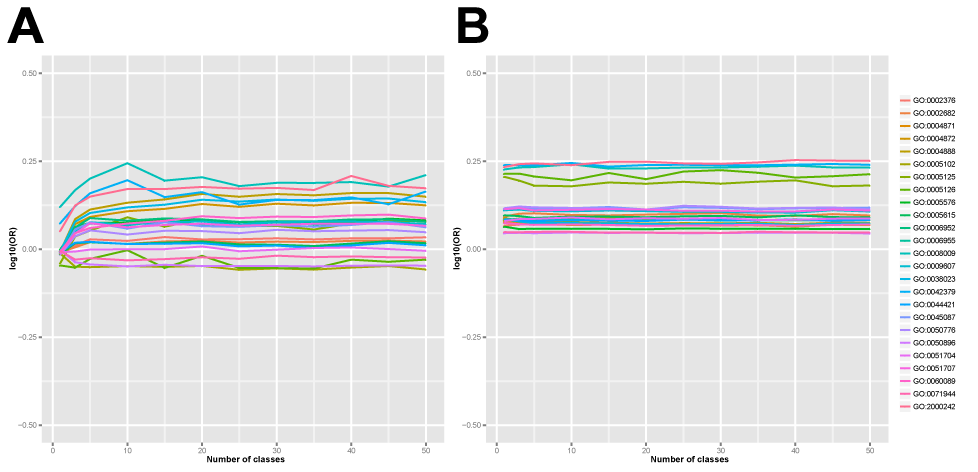

Supplement: Figure S4 — Performance of “permutation in quantiles” in functional category enrichment analysis. The mean (A) and s.d. (B) of each significant GO term in Group 1 was generated by 1000 replicates of permutation with varied number of classes delimited by the factor of dN/dS. The mean and s.d. of permutated OR distribution is not altered much when the number of class is larger than 15. (TIF) [file pone.0061280.s004.tif]

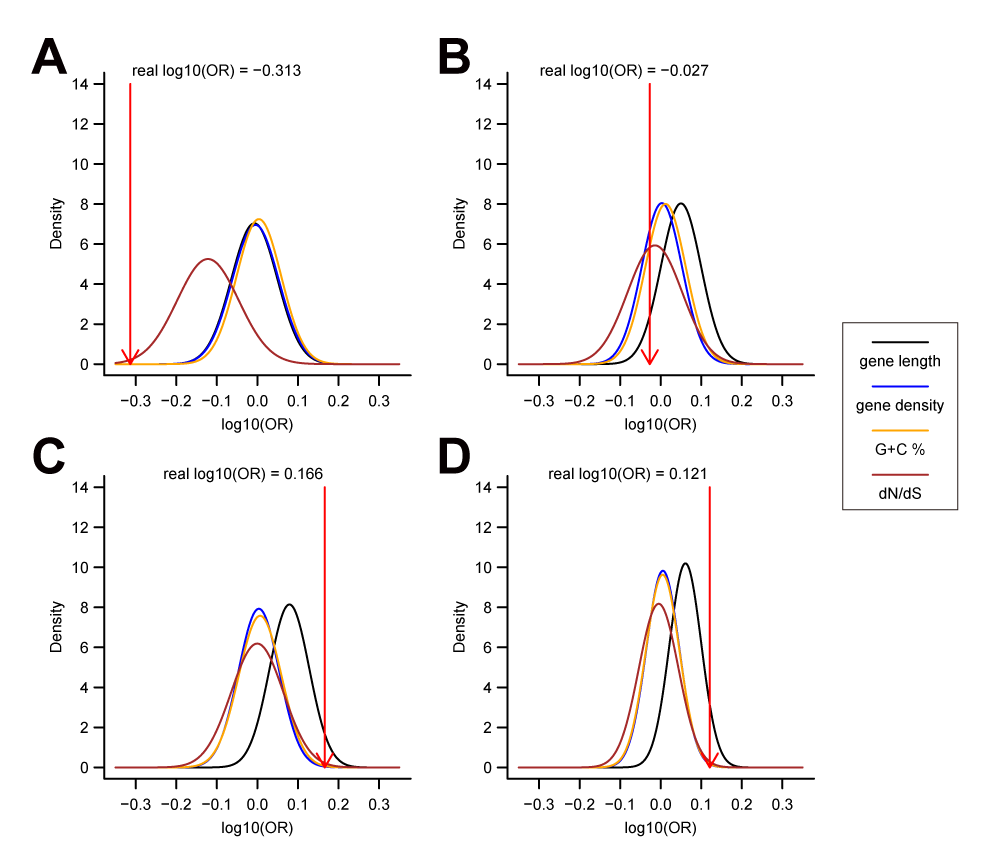

Supplement: Figure S5 — The observed under-representation of brain expression in Group 1 could not be accounted for all the four genomic characteristics, based on cDNA microarray dataset. The permutated OR distributions were generated by 1000 replicates after controlling gene length (black), gene density (blue), GC composition (yellow), and dN/dS (brown) for Group 1 to 4 positively selected genes (A–D). Group 1's real OR is significant smaller than expected by chance and it departs from all of the four permutated distributions. Although the real ORs of Group 3 and 4 fall within the 95% confidence interval after controlling gene length, they are larger than the averages of all the four permutated distributions. (TIF) [file pone.0061280.s005.tif]
