# Supplementary material for: Recent Adaptive Events in Human Brain Revealed by Meta-Analysis of Positively Selected Genes
Source: PLoS One. 2013 Apr 9;8(4):e61280. doi: 10.1371/journal.pone.0061280 (PMC3622023; doi:10.1371/journal.pone.0061280)
Supplement: Table S1 — Summary of grouped positively-selected genes by different approaches. (DOCX) [file pone.0061280.s006.docx]

**Table S1.** Summary of grouped positively-selected genes by different approaches

|  | **# of Genes** | **PubMed ID of References** |
| --- | --- | --- |
| **Group 1** |  |  |
|  | 39 | 15869325 |
|  | 304 | 16237444 |
|  | 588 | 16136131 |
|  | 106 | 14671302 |
|  | 113 | 17449636 |
|  | 54 | 18670650 |
|  | 77 | 16683019 |
|  | 1141 | All |
|  |  |  |
| **Group 2** |  |  |
|  | 4 | 16251466 |
|  | 174 | 16251465 |
|  | 385 | 16825663 |
|  | 163 | 17542651 |
|  | 430 | 20448178 |
|  | 1033 | All |
|  |  |  |
| **Group 3** |  |  |
|  | 156 | 12466284 |
|  | 31 | 18667002 |
|  | 602 | 18320033 |
|  | 320 | 20416085 |
|  | 1058 | All |
|  |  |  |
| **Group 4** |  |  |
|  | 15 | 16255080 |
|  | 119 | 16371466 |
|  | 19 | 16494531 |
|  | 290 | 17579516 |
|  | 718 | 17356696 |
|  | 43 | 16845142 |
|  | 16 | 17943131 |
|  | 364 | 17943122 |
|  | 164 | 19307593 |
|  | 169 | 19924308 |
|  | 1660 | All |
|  |  |  |
| **Composite** |  |  |
|  | 179 | 20056855 |
|  |  |  |
