# Supplementary material for: Recent Adaptive Events in Human Brain Revealed by Meta-Analysis of Positively Selected Genes
Source: PLoS One. 2013 Apr 9;8(4):e61280. doi: 10.1371/journal.pone.0061280 (PMC3622023; doi:10.1371/journal.pone.0061280)
Supplement: Table S2 — Enriched GO terms in different groups of human positively-selected genes. (DOCX) [file pone.0061280.s007.docx]

**Table S2.** Enriched GO terms in different groups of human positively-selected genes

|  | GO Term | Accession | Category | P-value | Corrected P-value |
| --- | --- | --- | --- | --- | --- |
| Group 1 |  |  |  |  |  |
|  | Immune response | GO:0006955 | BP | 2.38E-10 | 6.72E-07 |
|  | Defense response | GO:0006952 | BP | 1.07E-09 | 2.01E-06 |
|  | Regulation of transposition | GO:0010528 | BP | 3.93E-06 | 0.001846 |
|  | Negative regulation of transposition | GO:0010529 | BP | 3.93E-06 | 0.001846 |
|  | Innate immune response | GO:0045087 | BP | 1.07E-05 | 0.003776 |
|  | Immune system process | GO:0002376 | BP | 1.42E-05 | 0.004435 |
|  | Transposition | GO:0032196 | BP | 2.18E-05 | 0.006474 |
|  | Response to other organism | GO:0051707 | BP | 2.53E-05 | 0.007131 |
|  | Response to biotic stimulus | GO:0009607 | BP | 4.73E-05 | 0.012695 |
|  | T cell activation via T cell receptor contact with antigen bound to MHC molecule on antigen presenting cell | GO:0002291 | BP | 9.67E-05 | 0.024776 |
|  | Regulation of immune system process | GO:0002682 | BP | 1.01E-04 | 0.024837 |
|  | Regulation of immune response | GO:0050776 | BP | 1.13E-04 | 0.026496 |
|  | Multi-organism process | GO:0051704 | BP | 1.65E-04 | 0.037205 |
|  | Negative regulation of reproductive process | GO:2000242 | BP | 2.06E-04 | 0.044607 |
|  | Response to stimulus | GO:0050896 | BP | 2.48E-04 | 0.048174 |
|  | Extracellular region | GO:0005576 | CC | 1.91E-13 | 1.08E-09 |
|  | Extracellular space | GO:0005615 | CC | 5.45E-06 | 0.002196 |
|  | Extracellular region part | GO:0044421 | CC | 1.33E-05 | 0.004413 |
|  | Cell periphery | GO:0071944 | CC | 2.32E-04 | 0.048174 |
|  | Receptor activity | GO:0004872 | MF | 9.42E-09 | 1.33E-05 |
|  | Transmembrane signaling receptor activity | GO:0004888 | MF | 5.22E-07 | 0.000589 |
|  | Signaling receptor activity | GO:0038023 | MF | 1.07E-06 | 0.001004 |
|  | Signal transducer activity | GO:0004871 | MF | 2.81E-06 | 0.001846 |
|  | Molecular transducer activity | GO:0060089 | MF | 2.81E-06 | 0.001846 |
|  | Chemokine activity | GO:0008009 | MF | 3.57E-06 | 0.001846 |
|  | Chemokine receptor binding | GO:0042379 | MF | 3.81E-06 | 0.001846 |
|  | Cytokine activity | GO:0005125 | MF | 5.31E-06 | 0.002196 |
|  | Cytokine receptor binding | GO:0005126 | MF | 6.33E-06 | 0.002378 |
|  | Receptor binding | GO:0005102 | MF | 2.41E-04 | 0.048174 |
|  |  |  |  |  |  |
| Group 3 |  |  |  |  |  |
|  | Forebrain development | GO:0030900 | BP | 3.30E-06 | 0.005023 |
|  | Response to stimulus | GO:0050896 | BP | 8.90E-06 | 0.009034 |
|  | Telencephalon development | GO:0021537 | BP | 1.22E-05 | 0.009863 |
|  | Small GTPase mediated signal Transduction | GO:0007264 | BP | 1.42E-05 | 0.009863 |
|  | Cerebral cortex development | GO:0021987 | BP | 1.52E-05 | 0.009863 |
|  | Pallium development | GO:0021543 | BP | 1.62E-05 | 0.009863 |
|  | Subpallium development | GO:0021544 | BP | 2.05E-05 | 0.01028 |
|  | Nervous system development | GO:0007399 | BP | 2.21E-05 | 0.01028 |
|  | Cellular response to stimulus | GO:0051716 | BP | 2.65E-05 | 0.01028 |
|  | Regulation of cellular process | GO:0050794 | BP | 3.01E-05 | 0.01028 |
|  | Multicellular organismal process | GO:0032501 | BP | 6.75E-05 | 0.017146 |
|  | Signal transduction | GO:0007165 | BP | 6.81E-05 | 0.017146 |
|  | Ras protein signal transduction | GO:0007265 | BP | 7.04E-05 | 0.017146 |
|  | Cell communication | GO:0007154 | BP | 8.38E-05 | 0.018747 |
|  | Brain development | GO:0007420 | BP | 9.45E-05 | 0.018747 |
|  | Response to ammonium ion | GO:0060359 | BP | 9.76E-05 | 0.018747 |
|  | Urogenital system development | GO:0001655 | BP | 1.07E-04 | 0.019657 |
|  | Regulation of biological process | GO:0050789 | BP | 1.61E-04 | 0.025197 |
|  | Signaling | GO:0023052 | BP | 2.29E-04 | 0.033258 |
|  | Response to low-density lipoprotein particle stimulus | GO:0055098 | BP | 2.70E-04 | 0.035755 |
|  | Cholesterol storage | GO:0010878 | BP | 2.90E-04 | 0.037583 |
|  | Lipid storage | GO:0019915 | BP | 2.97E-04 | 0.037674 |
|  | Central nervous system development | GO:0007417 | BP | 3.18E-04 | 0.039567 |
|  | Kidney development | GO:0001822 | BP | 3.74E-04 | 0.045342 |
|  | Neurogenesis | GO:0022008 | BP | 3.80E-04 | 0.045342 |
|  | Biological regulation | GO:0065007 | BP | 3.89E-04 | 0.045493 |
|  | Cytoskeleton organization | GO:0007010 | BP | 4.51E-04 | 0.049982 |
|  | Synapse | GO:0045202 | CC | 1.49E-08 | 9.07E-05 |
|  | Synapse part | GO:0044456 | CC | 7.27E-08 | 0.000221 |
|  | Terminal button | GO:0043195 | CC | 2.57E-07 | 0.000522 |
|  | Axon terminus | GO:0043679 | CC | 8.89E-06 | 0.009034 |
|  | Axon part | GO:0033267 | CC | 2.26E-05 | 0.01028 |
|  | Neuron projection terminus | GO:0044306 | CC | 2.42E-05 | 0.01028 |
|  | Neuron projection | GO:0043005 | CC | 2.93E-05 | 0.01028 |
|  | Dendrite | GO:0030425 | CC | 3.04E-05 | 0.01028 |
|  | Synaptic membrane | GO:0097060 | CC | 5.37E-05 | 0.015971 |
|  | Cell junction | GO:0030054 | CC | 6.11E-05 | 0.016911 |
|  | Cell projection | GO:0042995 | CC | 9.32E-05 | 0.018747 |
|  | Dendritic spine | GO:0043197 | CC | 9.55E-05 | 0.018747 |
|  | Neuron spine | GO:0044309 | CC | 9.55E-05 | 0.018747 |
|  | Cytoplasm | GO:0005737 | CC | 1.56E-04 | 0.024989 |
|  | Cell projection part | GO:0044463 | CC | 1.80E-04 | 0.0274 |
|  | Cytoskeleton | GO:0005856 | CC | 2.02E-04 | 0.029945 |
|  | Cytosol | GO:0005829 | CC | 4.38E-04 | 0.049412 |
|  | Cytoskeletal protein binding | GO:0008092 | MF | 4.90E-05 | 0.015702 |
|  | Protein binding | GO:0005515 | MF | 5.51E-05 | 0.015971 |
|  | Ionotropic glutamate receptor activity | GO:0004970 | MF | 9.85E-05 | 0.018747 |
|  | Glutamate receptor activity | GO:0008066 | MF | 1.18E-04 | 0.021074 |
|  | Guanyl-nucleotide exchange factor activity | GO:0005085 | MF | 1.27E-04 | 0.022009 |
|  | Extracellular-glutamate-gated ion channel activity | GO:0005234 | MF | 1.39E-04 | 0.023426 |
|  | GTPase regulator activity | GO:0030695 | MF | 1.55E-04 | 0.024989 |
|  | Kainate selective glutamate Receptor activity | GO:0015277 | MF | 2.53E-04 | 0.035755 |
|  | Excitatory extracellular Ligand-gated ion channel activity | GO:0005231 | MF | 2.62E-04 | 0.035755 |
|  | Nucleoside-triphosphatase regulator activity | GO:0060589 | MF | 2.70E-04 | 0.035755 |
|  | Extracellular ligand-gated ion channel activity | GO:0005230 | MF | 4.19E-04 | 0.048103 |
|  |  |  |  |  |  |
| Group 4 |  |  |  |  |  |
|  | Ethanol oxidation | GO:0006069 | BP | 1.49E-07 | 0.000365 |
|  | Ethanol metabolic process | GO:0006067 | BP | 1.01E-06 | 0.00093 |
|  | Regulation of cell communication | GO:0010646 | BP | 1.97E-06 | 0.001313 |
|  | Primary alcohol metabolic process | GO:0034308 | BP | 4.41E-06 | 0.002159 |
|  | Regulation of localization | GO:0032879 | BP | 5.79E-06 | 0.002361 |
|  | Signaling | GO:0023052 | BP | 1.05E-05 | 0.004037 |
|  | Transmission of nerve impulse | GO:0019226 | BP | 1.17E-05 | 0.004284 |
|  | Cell communication | GO:0007154 | BP | 1.73E-05 | 0.006018 |
|  | Multicellular organismal signaling | GO:0035637 | BP | 1.80E-05 | 0.006018 |
|  | Wound healing | GO:0042060 | BP | 5.50E-05 | 0.01494 |
|  | Localization | GO:0051179 | BP | 6.94E-05 | 0.017561 |
|  | Multicellular organismal process | GO:0032501 | BP | 9.92E-05 | 0.023481 |
|  | Synaptic transmission | GO:0007268 | BP | 1.18E-04 | 0.025171 |
|  | Regulation of biological quality | GO:0065008 | BP | 1.65E-04 | 0.032722 |
|  | Cellular response to stimulus | GO:0051716 | BP | 2.33E-04 | 0.040651 |
|  | Cell projection | GO:0042995 | CC | 2.38E-08 | 8.74E-05 |
|  | Plasma membrane | GO:0005886 | CC | 2.91E-07 | 0.00053 |
|  | Cell periphery | GO:0071944 | CC | 3.65E-07 | 0.00053 |
|  | Plasma membrane part | GO:0044459 | CC | 7.98E-07 | 0.000837 |
|  | Synapse | GO:0045202 | CC | 1.70E-06 | 0.001313 |
|  | Synapse part | GO:0044456 | CC | 1.96E-06 | 0.001313 |
|  | Cell part | GO:0044464 | CC | 2.52E-06 | 0.001463 |
|  | Cell | GO:0005623 | CC | 2.59E-06 | 0.001463 |
|  | Cell projection part | GO:0044463 | CC | 4.94E-06 | 0.002264 |
|  | Lamellipodium membrane | GO:0031258 | CC | 2.02E-05 | 0.006438 |
|  | Cytoplasmic vesicle part | GO:0044433 | CC | 2.37E-05 | 0.007235 |
|  | Cytoplasm | GO:0005737 | CC | 4.41E-05 | 0.012447 |
|  | Cytoskeleton | GO:0005856 | CC | 1.20E-04 | 0.025171 |
|  | Protein binding | GO:0005515 | MF | 1.11E-08 | 8.12E-05 |
|  | Alcohol dehydrogenase (NAD) activity | GO:0004022 | MF | 4.33E-07 | 0.00053 |
|  | Binding | GO:0005488 | MF | 3.34E-06 | 0.001749 |
|  | Protein domain specific binding | GO:0019904 | MF | 5.52E-06 | 0.002361 |
|  | Alcohol dehydrogenase activity, zinc-dependent | GO:0004024 | MF | 3.24E-05 | 0.009508 |
|  | Calmodulin binding | GO:0005516 | MF | 6.29E-05 | 0.016469 |
|  | Glutamate receptor activity | GO:0008066 | MF | 7.46E-05 | 0.018238 |
|  | Retinoid binding | GO:0005501 | MF | 1.04E-04 | 0.023938 |
|  | Substrate-specific transporter activity | GO:0022892 | MF | 1.16E-04 | 0.025171 |
|  | Ion transmembrane transporter activity | GO:0015075 | MF | 1.45E-04 | 0.029574 |
|  | Isoprenoid binding | GO:0019840 | MF | 1.94E-04 | 0.037532 |
|  | SH3 domain binding | GO:0017124 | MF | 2.10E-04 | 0.039135 |
|  | Protein tyrosine kinase activity | GO:0004713 | MF | 2.13E-04 | 0.039135 |
|  | Lipid binding | GO:0008289 | MF | 2.20E-04 | 0.039373 |
|  |  |  |  |  |  |

**CC:** Cellular Component; **BP:** Biological Process; **MF:** Molecular Function
