# Supplementary material for: Recent Adaptive Events in Human Brain Revealed by Meta-Analysis of Positively Selected Genes
Source: PLoS One. 2013 Apr 9;8(4):e61280. doi: 10.1371/journal.pone.0061280 (PMC3622023; doi:10.1371/journal.pone.0061280)
Supplement: Table S3 — Odds ratios of brain-biased expression enrichment analysis for each groups of positively-selected genes. (DOCX) [file pone.0061280.s008.docx]

**Table S3.** Odds ratios of brain-biased expression enrichment analysis for each groups of positively-selected genes.

|  | mRNA-SEQ | cDNA microarray |
| --- | --- | --- |
| Group 1 | 0.58 | 0.49 |
| Group 2 | 1.01 | 0.94 |
| Group 3 | 1.39 | 1.47 |
| Group 4 | 1.47 | 1.32 |
| Group composite | 1.69 | 2.07 |
