# Supplementary material for: Recent Adaptive Events in Human Brain Revealed by Meta-Analysis of Positively Selected Genes
Source: PLoS One. 2013 Apr 9;8(4):e61280. doi: 10.1371/journal.pone.0061280 (PMC3622023; doi:10.1371/journal.pone.0061280)
Supplement: Text S2 — The detailed description of identification approaches for 27 literatures identifying human positively selected genes. (DOCX) [file pone.0061280.s010.docx]

**Text S2. The detailed description of identification approaches for 27 literatures identifying human positively selected genes.** Genes with unique Entrez Gene ID annotation were collected into our list of positively-selected genes.

**2002**

Interrogating a high-density SNP map for signatures of natural selection [[1](#_ENREF_1)]

The F_ST_ was first calculated to evaluate the inbreeding coefficient between different subpopulations, and then the genes containing SNPs with F_ST_ >0.45 in autosomes and F_ST_ >0.59 in chromosome X were collected.

**2003**

Inferring nonneutral evolution from human-chimp-mouse orthologous gene trios [[2](#_ENREF_2)]

The nucleotide substitution sites of human and chimpanzee were analyzed by comparing against mouse as the out-group, and then the likelihood ratio test based on dN/dS was used to identify genes under positive selection. The result was filtered by P-value <0.01 in the human lineage.

**2005**

Genomic scans for selective sweeps using SNP data [[3](#_ENREF_3)]

The likelihood ratio of neutral and hitchhiking models in a genomic region was tested by using site frequency spectrum and spatial information based on Seattle SNP, and then the result was filtered by P-value <0.05.

A scan for positively selected genes in the genomes of humans and chimpanzees [[4](#_ENREF_4)]

The orthologous groups between human and chimpanzee were aligned, and then the codon-based likelihood method were used to identify genes under positive selection. The result was filtered by P-value <0.05.

Natural selection on protein-coding genes in the human genome [[5](#_ENREF_5)]

The orthologous groups between human and chimpanzee were aligned and further applied by advanced McDonald-Kreitman method to identify genes under positive selection, and then the genes with continuous positive signals with 95% credibility were collected.

A haplotype map of the human genome [[6](#_ENREF_6)]

The long-range haplotype test was applied to identify the genomic regions containing high frequency alleles with long-range LD, and then the regions were filtered by P-value <1e-5.

Initial sequence of the chimpanzee genome and comparison with the human genome [[7](#_ENREF_7)]

The orthologous groups between human and chimpanzee were aligned, and then the branch-site K_a_/K_i_ was calculated. The result was filtered by K_a_/K_i_ >1.

Genomic regions exhibiting positive selection identified from dense genotype data [[8](#_ENREF_8)]

A method to identify contiguous regions of Tajima’s D reduction was applied to find selective sweeps between subpopulations, and then the genes locating within these regions were collected.

**2006**

Global landscape of recent inferred Darwinian selection for Homo sapiens [[9](#_ENREF_9)]

An LD decay test primarily based on identification of significant LD difference surrounding a given SNP was used in multiple subpopulations, and the genes identified in all subpopulations were collected.

A map of recent positive selection in the human genome [[10](#_ENREF_10)]

An iHS test based on Extended Haplotype Homozygosity (EHH) was applied to detect the signal of long haplotype indicating selective sweeps, by using all SNPs with minor allele frequency >5% as the input. The genes with strongest signals that are listed in the article were collected.

Genomic signatures of positive selection in humans and the limits of outlier approaches [[11](#_ENREF_11)]

The Tajima’s D of each gene in different subpopulations was calculated, and then the genes with Tajima’s D locating in the 1% of the empirical distribution in each subpopulation were collected.

A whole genome long-range haplotype (WGLRH) test for detecting imprints of positive selection in human populations [[12](#_ENREF_12)]

A long-range haplotype test based on Gabriel’s D with the four-gamete test was applied, and then the regions with corrected P-value <0.05 were collected.

Positive selection, relaxation, and acceleration in the evolution of the human and chimp genome [[13](#_ENREF_13)]

The orthologous groups among five mammalian genomes (human, chimpanzee, mouse, rat, and dog) were aligned and the branch-site likelihood method was applied to identify genes under positive selection, and then the result was filtered by corrected P-value <0.05 in Test II.

**2007**

A new approach for using genome scans to detect recent positive selection in the human genome [[14](#_ENREF_14)]

The genomic regions with decay of EHH between different subpopulations were identified, and then the regions with strong signals in combined Perlegen and HapMap data were collected.

A practical genome scan for population-specific strong selective sweeps that have reached fixation [[15](#_ENREF_15)]

The EHH patterns between different subpopulations were calculated, and then the regions with both rMHH <0.05 and rHH <0.3, corresponding to approximately 90% detection power, were collected.

Genome-wide detection and characterization of positive selection in human populations [[16](#_ENREF_16)]

An advanced long-range haplotype test, together with iHS and cross-population EHH test, was applied. The regions able to pass all three tests and with no similar events found in simulation data were collected.

More genes underwent positive selection in chimpanzee evolution than in human evolution [[17](#_ENREF_17)]

The nucleotide substitution sites of human and chimpanzee were analyzed by comparing against macaque as the out-group, and then the branch-site likelihood method was used to identify genes under positive selection. The result was filtered by P-value <0.05 in the human lineage.

Localizing recent adaptive evolution in the human genome [[18](#_ENREF_18)]

The composite likelihood ratio test was applied to detect selective sweep by comparing the SFS in a genomic window to the SFS of the rest of the genome, and then the regions with P-value <1E-5 were collected.

A second generation human haplotype map of over 3.1 million SNPs [[19](#_ENREF_19)]

A combination of long-range haplotype test and iHS test was used on HapMap II data. The long-range haplotype test and the iHS test followed the procedure in [[16](#_ENREF_16)] and [[10](#_ENREF_10)], respectively.

**2008**

Patterns of positive selection in six mammalian genomes [[20](#_ENREF_20)]

The orthologous groups among six mammalian genomes (human, chimpanzee, macaque, mouse, rat, and dog) were aligned and the branch-site and clade-site likelihood method was applied to identify genes under positive selection. The result was filtered by P-value <0.05 in the human lineage.

Identification of local selective sweeps in human populations since the exodus from Africa [[21](#_ENREF_21)]

The F_ST_ and length of extended haplotype block between different subpopulations was calculated, and then the regions with combined P-value <0.01 were collected.

Identifying selected regions from heterozygosity and divergence using a light-coverage genomic dataset from two human populations [[22](#_ENREF_22)]

The arrayed F_ST_ and local heterozygosity between different subpopulations was calculated, and then the regions with λ(H_EA_) <3.8e-5, λ(H_AA_) <6.4e-4 and λ(S^2^F_ST_) <4.6e-4, corresponding to 95% detection power, were collected.

**2009**

Signals of recent positive selection in a worldwide sample of human populations [[23](#_ENREF_23)]

The iHS and XP-EHH tests were calculated based on the population genetic data from eight subpopulations. The genes under positive selection were extracted from the top 20 regions of two tests.

Genetic variation and recent positive selection in worldwide human populations: evidence from nearly 1 million SNPs [[24](#_ENREF_24)]

A modified lnRSB test based on the extended haplotype homozygosity was applied on the genotype date from seven geographic regions. The genes were extracted from the top 100 candidate regions in each subpopulation.

**2010**

A composite of multiple signals distinguishes causal variants in regions of positive selection [[25](#_ENREF_25)]

A composite method that integrates multiple signatures of recent positive selection, including long haplotypes, high-frequency derived alleles, and highly differentiated alleles, was used. The genes were then extracted from the genomic regions identified in HapMap II data.

A draft sequence of the Neandertal genome [[26](#_ENREF_26)]

A population genetic method was carried out by scanning the genomic regions which has fewer Neandertal-shared alleles in modern human than expected, and the genes under positive selection were extracted from the top 20 candidate regions.

Fine-scale detection of population-specific linkage disequilibrium using haplotype entropy in the human genome [[27](#_ENREF_27)]

The degree of genetic diversity was measured by using the entropy of haplotype frequency, and the entropy difference between CEU and YRI was calculated in a genomic window scan.

**Supplementary References**

1. Akey JM, Zhang G, Zhang K, Jin L, Shriver MD (2002) Interrogating a High-Density SNP Map for Signatures of Natural Selection. Genome Res 12: 1805-1814.

2. Clark AG, Glanowski S, Nielsen R, Thomas PD, Kejariwal A, et al. (2003) Inferring Nonneutral Evolution from Human-Chimp-Mouse Orthologous Gene Trios. Science 302: 1960-1963.

3. Nielsen R, Williamson S, Kim Y, Hubisz MJ, Clark AG, et al. (2005) Genomic scans for selective sweeps using SNP data. Genome Res 15: 1566-1575.

4. Nielsen R, Bustamante C, Clark AG, Glanowski S, Sackton TB, et al. (2005) A Scan for Positively Selected Genes in the Genomes of Humans and Chimpanzees. PLoS Biol 3: e170.

5. Bustamante CD, Fledel-Alon A, Williamson S, Nielsen R, Hubisz MT, et al. (2005) Natural selection on protein-coding genes in the human genome. Nature 437: 1153-1157.

6. Consortium TIH (2005) A haplotype map of the human genome. Nature 437: 1299-1320.

7. Consortium TCSaA (2005) Initial sequence of the chimpanzee genome and comparison with the human genome. Nature 437: 69-87.

8. Carlson CS, Thomas DJ, Eberle MA, Swanson JE, Livingston RJ, et al. (2005) Genomic regions exhibiting positive selection identified from dense genotype data. Genome Res 15: 1553-1565.

9. Wang ET, Kodama G, Baldi P, Moyzis RK (2006) Global landscape of recent inferred Darwinian selection for Homo sapiens. Proc Natl Acad Sci U S A 103: 135-140.

10. Voight BF, Kudaravalli S, Wen X, Pritchard JK (2006) A Map of Recent Positive Selection in the Human Genome. PLoS Biol 4: e72.

11. Kelley JL, Madeoy J, Calhoun JC, Swanson W, Akey JM (2006) Genomic signatures of positive selection in humans and the limits of outlier approaches. Genome Res 16: 980-989.

12. Zhang C, Bailey DK, Awad T, Liu G, Xing G, et al. (2006) A whole genome long-range haplotype (WGLRH) test for detecting imprints of positive selection in human populations. Bioinformatics 22: 2122-2128.

13. Arbiza L, Dopazo J, Dopazo H (2006) Positive Selection, Relaxation, and Acceleration in the Evolution of the Human and Chimp Genome. PLoS Comput Biol 2: e38.

14. Tang K, Thornton KR, Stoneking M (2007) A New Approach for Using Genome Scans to Detect Recent Positive Selection in the Human Genome. PLoS Biol 5: e171.

15. Kimura R, Fujimoto A, Tokunaga K, Ohashi J (2007) A Practical Genome Scan for Population-Specific Strong Selective Sweeps That Have Reached Fixation. PLoS One 2: e286.

16. Sabeti PC, Varilly P, Fry B, Lohmueller J, Hostetter E, et al. (2007) Genome-wide detection and characterization of positive selection in human populations. Nature 449: 913-918.

17. Bakewell MA, Shi P, Zhang J (2007) More genes underwent positive selection in chimpanzee evolution than in human evolution. Proc Natl Acad Sci U S A 104: 7489-7494.

18. Williamson SH, Hubisz MJ, Clark AG, Payseur BA, Bustamante CD, et al. (2007) Localizing Recent Adaptive Evolution in the Human Genome. PLoS Genet 3: e90.

19. Consortium TIH (2007) A second generation human haplotype map of over 3.1 million SNPs. Nature 449: 851-861.

20. Kosiol C, Vinar T, Fonseca RRd, Hubisz MJ, Bustamante CD, et al. (2008) Patterns of Positive Selection in Six Mammalian Genomes. PLoS Genet 4: e1000144.

21. Johansson A, Gyllensten U (2008) Identification of local selective sweeps in human populations since the exodus from Africa. Hereditas 145: 126-137.

22. Oleksyk TK, Zhao K, Vega FMDL, Gilbert DA, O'Brien SJ, et al. (2008) Identifying Selected Regions from Heterozygosity and Divergence Using a Light-Coverage Genomic Dataset from Two Human Populations. PLoS One 3: e1712.

23. Pickrell JK, Coop G, Novembre J, Kudaravalli S, Li JZ, et al. (2009) Signals of recent positive selection in a worldwide sample of human populations. Genome Res 19: 826-837.

24. Herraez DL, Bauchet M, Tang K, Theunert C, Pugach I, et al. (2009) Genetic Variation and Recent Positive Selection in Worldwide Human Populations: Evidence from Nearly 1 Million SNPs. PLoS One 4: e7888.

25. Grossman SR, Shylakhter I, Karlsson EK, Byrne EH, Morales S, et al. (2010) A Composite of Multiple Signals Distinguishes Causal Variants in Regions of Positive Selection. Science 327: 883-886.

26. Green RE, Krause J, Briggs AW, Maricic T, Stenzel U, et al. (2010) A Draft Sequence of the Neandertal Genome. Science 328: 710-722.

27. Mizuno H, Atwal G, Wang H, Levine AJ, Vazquez A (2010) Fine-scale detection of population-specific linkage disequilibrium using haplotype entropy in the human genome. BMC Genet 11: 27.
